# Analyzing intra-abdominal pressures and outcomes in patients undergoing emergency laparotomy

**DOI:** 10.4103/0974-2700.70747

**Published:** 2010

**Authors:** Shehtaj Khan, Akshay Kumar Verma, Syed Moied Ahmad, Reyaz Ahmad

**Affiliations:** Department of Surgery, J. N. Medical College, AMU, Aligarh-202002, Uttar Pradesh, India; 1 Department of Anesthesia, J. N. Medical College, AMU, Aligarh-202002, Uttar Pradesh, India

**Keywords:** Abdominal compartment syndrome, intra-abdominal hypertension, intra-abdominal pressure

## Abstract

**Background:**

Studies have documented the impact of intra-abdominal hypertension (IAH) on virtually every organ. However, it still remains strangely underdiagnosed. The aims of the study were to assess, in patients undergoing emergency laparotomy, whether intra-abdominal pressure (IAP) is an independent predictor of morbidity and mortality, to evaluate the effects of IAH, and to identify hidden cases of abdominal compartment syndrome (ACS).

**Materials and Methods:**

The study comprised 197 patients undergoing emergency laparotomy. IAP was measured preoperatively and then postoperatively at 0, 6, and 24 hours. Duration of hospital stay, occurrence of burst abdomen, and mortality were noted as outcomes.

**Results:**

At admission, incidence of IAH was 80%. No significant association was found between IAP and occurrence of burst abdomen (*P* > 0.1). IAP was found to be a significant predictor of mortality in patients undergoing laparotomy (*P* < 0.001). Elevated IAP was found to affect all the organ systems adversely. The incidence of post-op ACS was 3.05% in the general population and 13.16% in trauma patients. The mortality rate for this subgroup was 100%.

**Conclusions:**

IAP is a significant predictor of mortality in patients undergoing laparotomy. IAH has detrimental effects on various organ systems. A more frequent monitoring with prompt decompression may be helpful in decreasing the mortality rate. Further studies are required to establish a screening protocol in patients undergoing laparotomy to detect and manage cases of IAH and ACS.

## INTRODUCTION

The effect of the increased intra-abdominal pressure (IAP) in various organ systems has been studied over the past century.[1] Emerson first noted the cardiovascular morbidity and mortality associated with elevated IAP in 1911.[2] However, the recognition of abdomen as a compartment and the concept of intra-abdominal hypertension (IAH) resulting in abdominal compartment syndrome (ACS) have only recently received attention.

IAP is the pressure concealed within the abdominal cavity.[3] Although IAP can physiologically reach elevated values transiently up to 80 mm Hg (cough, Valsalva maneuver, weight lifting, etc.), these values cannot be tolerated for long periods.[4,5] Normal IAP is approximately 5–7 mm Hg in critically ill adults.[3] IAH is defined as an intra-abdominal pressure above 12 mm Hg.[3] The presence of IAH is associated with an 11-fold increase in mortality compared with patients without IAH.[6] The detrimental effects of IAH occur long before the manifestation of compartment syndrome. The ACS, therefore, should be viewed as the end result of a progressive, unchecked increase in IAP from a myriad of disorders that eventually leads to multiple organ dysfunction.[1]

Rapid progression of IAH leads to ACS, which is defined as an IAP greater than 20 mm Hg with at least one new organ system dysfunction/failure.[3] Elevated IAP produces multiple derangements in both intra- and extra-abdominal organs. While adverse effects on kidneys and lung have been well recognized, subsequent studies have documented an impact on virtually every organ except the adrenal glands.[7]

But there are always two sides to a coin, and IAP is no different. The beneficial effect of raised IAP has been reported in a study which found that intraperitoneal chemotherapy with increased IAP, in comparison with conventional IP or IV chemotherapy, improved the tumor accumulation and the antitumor effect of Cisplatin.[8]

Yet, despite the abundance of knowledge, IAH still remains strangely underdiagnosed. A national postal questionnaire in the United Kingdom reported that despite widespread awareness of IAH and the ACS, many intensive care units never measure the IAP.[9] Hence, it is high time that we open our eyes to this entity and protect our patients from its deleterious effects before it is too late.

The aims of this study were

1. to assess, in patients undergoing emergency laparotomy, whether IAP is an independent predictor of morbidity and mortality;
2. to evaluate the effects of IAH;
3. to identifying the hidden cases of ACS.

## MATERIALS AND METHODS

### Study design

This was a prospective observational study conducted in Department of General Surgery, J. N. Medical College, AMU, Aligarh, on patients attending Emergency Department of Surgery, over a period of 1 year and 10 months from January 2006 to October 2007.

Following cases were considered eligible for inclusion in the study:

1. age ≥ 18 years and
2. all the patients undergoing emergency laparotomy.

Following cases were excluded from the study:

1. pregnant patients and
2. patients in whom Foley’s catheterization was not possible.

### Method

A patient was included in the study only after a decision to operate upon him/her was taken. Patient particulars were noted along with the indication for surgery. Readings were taken preoperatively and then postoperatively at 0, 6, 24, and 72 hours. If IAP remained below 12 mm Hg, measurements were discontinued after 24 hours.

Parameters noted were

1. blood pressure,
2. pulse rate,
3. respiratory rate,
4. oxygen saturation (SpO_2_),
5. temperature,
6. urine output,
7. IAP,
8. duration of surgery,
9. per-op findings,
10. duration of hospital stay,
11. morbidity (burst abdomen), and
12. Mortality.

Following laboratory investigations were conducted:

1. blood urea and
2. serum creatinine.

### Measurement of intra-abdominal pressure

The abdominal pressure was indirectly determined by measuring urinary bladder pressure with a Foley’s catheter. Patient was catheterized with a 16-guage Foley’s catheter. The bladder was drained and then filled with 50 ml of sterile saline through the Foley’s catheter. The tubing of the collecting bag was clamped. The catheter was connected to a saline manometer. The symphysis pubis was the zero reference, and pressure was measured in centimeters of water at end-expiration. A conversion factor of 1.36 was used to convert the pressure into millimeter of Hg. The IAP measurements had some limitations in our study.

### Interpretation

#### Grading of intra-abdominal hypertension

1. Grade I: 12–15 mm Hg;
2. Grade II: 16–20 mm Hg;
3. Grade III: 21–25 mm Hg; and
4. Grade IV: >25 mm Hg.

The term “abdominal compartment syndrome” was used when IAH was associated with at least one newly developed organ system dysfunction.

In patients with ACS, the decision to proceed with decompressive laparotomy lays in the hands of the primary surgeon in-charge of the patient.

### Organ system derangement

#### Cardiovascular system

1. Blood pressure < 90 mm Hg systolic or
2. heart rate > 100/minute or
3. both of the above.

#### Respiratory system

1. Respiratory rate > 20/minute or
2. SpO_2_ < 90% or
3. the patient requires ventilatory support or
4. any two or all of the above.

#### Renal

1. Blood urea > 40 mg% or
2. serum creatinine > 1.2 mg% or
3. urine output < 25 ml/hour or
4. any two or all of the above.

### Statistical analysis

The data were analyzed using Statistical Package for Social Sciences (SPSS) for Windows Version 10.

To study the nature and dynamics of relationship between duration of stay of patient in the hospital and IAP, Carl Pearson’s product moment correlation was used. In order to investigate the predictive role of IAP as a determinant of duration of stay in hospital, linear regression analysis was conducted. Since our concern is to identify only IAP as a predictor, univariate regression with forced entry method was conducted. Goodness of fit analysis of variance (ANOVA) statistics was used to determine whether our regression model was significant and whether there was any significant change in the variance of a dependent variable due to the entry of an independent variable. The *t* value indicates the significance of coefficient of regression (β). Point bi-serial correlation was used to study the relationship between IAP and occurrence of burst abdomen and mortality. In order to study the predictive association of death and burst abdomen with different levels of abdominal pressure, binary logistic regression analysis was conducted. To see the effect of IAP on various organ systems at a particular point, independent samples *t*-test was used. McNemar statistics for change was used to analyze the effect of laparotomy/decompression on the state of the patient.

## RESULTS

A total of 208 patients were included in this study but 11 patients did not have the readings taken according to the requirements for data collection. Hence, a total of 197 patients comprised the study population. Of these, there were 149 men and 48 women. The mean age was 34.78±14.9 (range 18–85) years with the most common age of presentation being 18 years. Most of the patients were in the age group of 21–40 years.

Various indications for laparotomy were as follows:

1. Perforation peritonitis: 64%;
2. acute intestinal obstruction: 14%;
3. gunshot: 10%;
4. blunt trauma abdomen: 8%;
5. pyoperitoneum: 2% and
6. stab injury: 2%.

Trauma: non-trauma distribution = 19:81.

Pre-op IAP grading is as shown in Table 1. Pre-op IAH was seen in 80% of the patients.

**Table 1:** Pre-operative IAP grading

	No. of patients	Non-trauma	Trauma
No IAH	38	25	13
Grade I	52	43	9
Grade II	56	47	9
Grade III	36	30	6
Grade IV	15	14	1

IAP, INTRA-ABDOMINAL PRESSURE

Per-op findings in trauma patients were as follows:

1. Liver laceration: 9 (1 with associated splenic laceration); packing done in 4;
2. splenic laceration: 4; splenectomy done in all 4;
3. hollow viscera perforation: 23; primary closure in 17, exteriorization in 6; and
4. retro-peritoneal hematoma: 3 (2 with associated urethral injuries); packing in 1.

**Figure 1:**
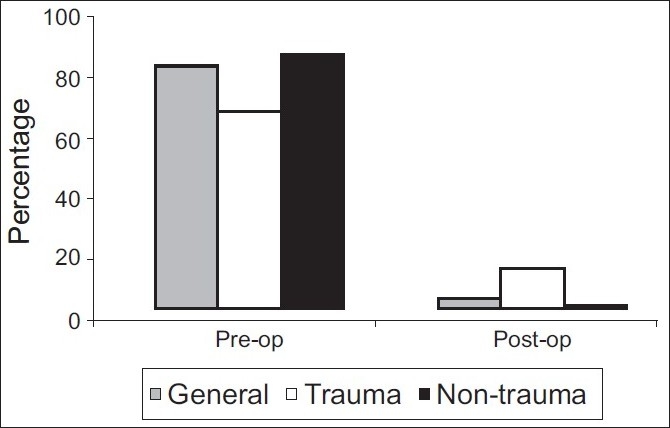
Incidence of IAH

No IAH was observed at 0 hours post-op. Post-op (6 hours) IAP grading is as shown in Table 2. Incidence of IAH is shown in Figure 1.

**Table 2:** Post-op (6 hours) IAP grading

	Non-trauma	Trauma	Total
No IAH	157	33	190
Grade I	0	0	0
Grade II	1	0	1
Grade III	0	3	3
Grade IV	1	2	3

IAH, INTRA-ABDOMINAL HYPERTENSION; IAP, INTRA-ABDOMINAL PRESSURE

### General population

1. Pre-op IAH: 80.20%;
2. IAH at 0 hours post-op: 0%;
3. IAH at 6 hours post-op: 3.55%; and
4. IAH at 24 hours post-op: 0%

### Trauma

1. Pre-op IAH: 65.79% and
2. IAH at 6 hours post-op: 13.16%.

### Non-trauma

1. Pre-op IAH: 83.65%; and
2. IAH at 6 hours post-op: 1.26%.

### Incidence of ACS

1. General population: 3.05%;
2. trauma: 13.16%; and
3. non-trauma: 0.63%.

Organ system dysfunction associated with IAH is shown in Table 3. Two patients were referred to higher center; hence, their survival/mortality could not be noted.

**Table 3:** Organ system dysfunction associated with IAH

	Pre-op IAH	Pre-op no IAH	Post-op IAH	Post-op no IAH
Renal dys	78	8	7	35
No renal dys	80	31	0	155
CVS dys	122	23	6	14
No CVS dys	36	16	1	173
Resp. dys	140	30	7	15
No resp. dys	18	9	0	172
Death	21	1	7	15
Survival	135	38	0	172
Burst abdomen	25	6	0	30
No burst abdomen	133	33	7	160

CVS, CARDIOVASCULAR SYSTEM; RESP., RESPIRATORY SYSTEM; DYS., DYSFUNCTION; IAH, INTRAABDOMINAL HYPERTENSION

**Graph 1:**
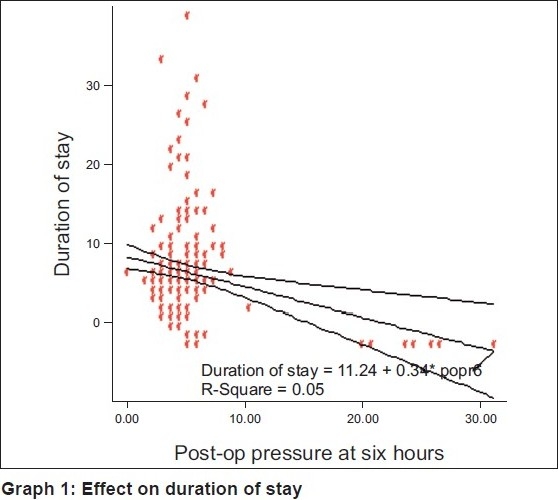
Effect on duration of stay

Pearson’s correlation between duration of stay and different readings of abdominal pressure as shown in Table 4 indicates that relationship between duration of stay and post-op pressures at 6 hours have significant negative relationship, i.e., if post-op pressure at 6 hours increases, duration of stay decreases. The respective *P* values for linear regression between IAP pre-op, postop at 0, 6, and 24 hours, and duration of stay were 0.410, 0.855, 0.001, and 0.129. This suggests that IAP post-op at 6 hours is a significant predictor of duration of stay. The model summary and coefficients are shown in Table 5. This table shows goodness of fit ANOVA to be highly significant. There is a significant change in the variance of duration of stay due to post-op pressure at 6 hours. Regression value for this variable is 0.234 that indicates appropriate predictability in the relationship between the duration of stay and post-op pressure at 6 hours. R^2^ value (0.055) indicates that it accounts for 5.5% of the total variance in duration of stay. β indicates nature of relationship between the two variables. Each unit increase in the level of post-op pressure at 6 hours will decrease the duration of stay by 0.342 days (provided the effect of all other factors is kept constant). Confidence intervals for β show that our findings are true to the entire population from which the sample has been taken (shown in Graph 1).

**Table 4:** Pearson’s correlation between duration of stay and abdominal pressure

	*r*	*P* value
Pre-op pressure	−0.059	0.410
Post-op pressure at 0 hour	−0.013	0.855
Post-op pressure at 6 hours	−0.234	0.001
Post-op pressure at 24 hours	0.112	0.129

**Table 5:** Model Summary and coefficients of relationship between post-op pressure (6 hours) and duration of stay

Predictor	*R*	*R*^2^	β	*t*	*P*	95% confidence interval for β
Post-op pressure	0.234	0.055	−0.342	−3.328	0.001	−0.545 to −0.139 constant = 11.238

If the population of this sample is well defined and all parameters are taken in a similar way, our β value and constant can be used for future interpretations. With the help of regression equation, future estimation of duration of stay for the prospective patients with similar conditions can be calculated.

Regression equation:

Y = β0 + β1X1 + β2X2 + … +βnXn + εi (for a multivariate hierarchical regression).

β - coefficient of regression, *X*- variable

Extrapolation for the present population:

duration of stay=11.238 + (−0.342 × post-op pressure at 6 hours).

When the analysis was repeated after dropping patients with raised pressure at 6 hours, *P* value was 0.307 which is nonsignificant. Point bi-serial correlation between occurrence of burst abdomen and IAP showed no significant results, but that between occurrence of death of patient and IAP is shown in Table 6. This table indicates that occurrence of death is positively associated with pre-op pressure (*P* = 0.005), post-op pressure at 0 hours (*P* = 0.002), and post-op pressure at 6 hours (0.000). Binary logistic regression between occurrence of burst abdomen and IAP showed non-significant value indicating that our model is not having any predictive value (*P* = 0.921, 0.0704, 0.406, 0.162 at pre-op, post-op 0, 6, 24 hours, respectively). Binary logistic regression between occurrence of death of patient and IAP indicates that our model is strong enough and acceptable to predict the relationship between occurrence of death and pre-op pressure as well as post-op pressures at 0 and 6 hours. However, a comparative view shows that odds of death are stronger as a result of increase in the post-op pressure at 6 hours in comparison to other two types of pressures.

**Table 6:** Point bi-serial correlation between occurrence of death and IAP

	Death	*P*
Pre-op pressure	0.201	0.005
Post-op pressure at 0 hours	0.219	0.002
Post-op pressure at 6 hours	0.572	0.000
Post-op pressure at 24 hours	0.070	0.350

### Effect of IAP on various organ systems

To see the effect of IAP on various organ systems at a particular point, our sample was bifurcated into two categories, i.e., those who had IAH and those who did not have IAH. Both the groups were subjected to the independent samples *t*-test to be compared on various physiological systems. There is significant detrimental effect of elevated IAP at 6 hours on all the recorded parameters as compared to normal IAP.

Changes in organ systems pre- and post-op are shown in Table 7. This table shows that there is significant change in all the systems after laparotomy [cardiovascular system (CVS) *P* < 0.001, respiratory system *P* < 0.02, renal functions *P* < 0.001].

**Table 7:** Changes in organ systems pre- and post-op

		Post-op 0 hours	
		Not deranged	Deranged
Pre-op CVS	Deranged	15(9.5)	107(67.7)
	Not deranged	7(4.4)	29(18.4)
Pre-op respiratory system	Deranged	22(13.9)	118(74.7)
	Not deranged	9(5.7)	9(5.7)
Pre-op renal functions	Deranged	25(15.8)	53(33.5)
	Not deranged	75(47.5)	5(3.2)

Figures IN PARENTHESIS ARE IN PERCENTAGE

### Mortality rate

**Figure 2:**
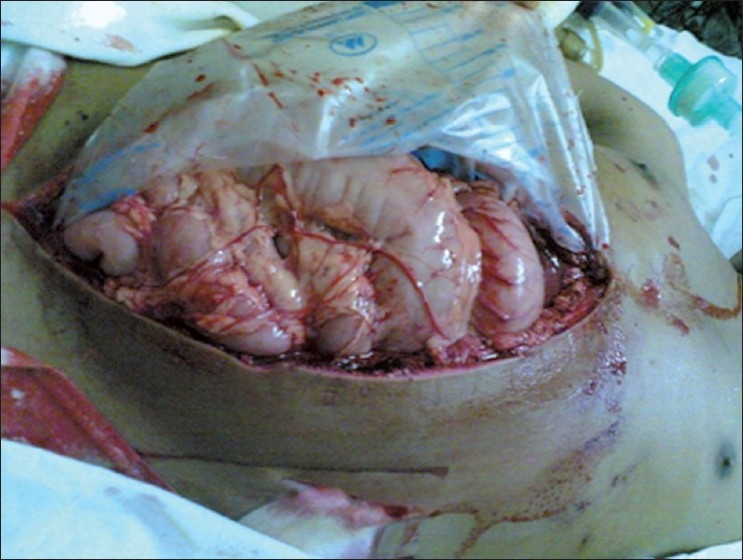
Decompression with laparotomy

Pre-op IAH: 13.2%; post-op ACS: 100% (three patients decompressed after diagnosis and abdomen left open, but none survived; Figure 2).

## DISCUSSION

Most of the studies on IAH and ACS analyze either trauma or ICU patients. Little has been reported on ACS in general surgical population. Our study population was a group of patients who underwent laparotomy for various indications which included traumatic as well as non-traumatic causes. There were 149 (76%) males and 48 (24%) females. A similar ratio was seen in the studies by Hong *et al*. (72% males) and Meldrum *et al*. (70% males), but Sugrue *et al*. and Cheatham *et al*. reported about 60% males in their study groups.[6,10–12] The mean ± SD (range) age in our study was 34.78±14.9 (range 18–85) years. Most of the studies report the mean age to be higher than what we observed. Cheatham *et al*. have reported a mean age of 51±19 years, Meldrum *et al*. 39±9 years, and Hong *et al*. 42 years.[10–12] Of the 197 patients, there were 38 (19%) trauma patients. This is in contrast to the study by Cheatham *et al*. who had 68% trauma patients in their study group.[11] This can be explained by the population selected for the study. Their patients were critically ill and required ICU care hence a predominance of trauma patients, while our sample included all those who underwent laparotomy, hence a predominance of general surgical patients.

In the trauma group, the injury mechanics was blunt in 15 (40%) and penetrating in 23 (60%) patients. Five (13%) patients underwent packing. Meldrum *et al*. reported 60% blunt injuries and packing in 67% cases.[10] This reflects the demographic variations in the study population. In developing countries, injuries due to assault are a major cause of trauma in patients. These are mostly gunshots or stab injuries. While in developed countries, road traffic accidents are the most common cause of trauma, hence higher incidence of blunt injuries. Also, the severity of injury is more due to high speed trauma there. Thus, lesser number of our patients required packing. The mean (SD) IAPs before and after laparotomies were 18 (4.8) mm Hg and 6 (1.7) mm Hg, respectively, in the patients who had IAH at admission. The mean (SD) IAPs in the study group of Sugrue *et al*. before and after decompressions were 16.6 (9.4) mm Hg and 10.3 (3.1) mm Hg, respectively. Meldrum *et al*. reported higher values of IAP (SD) pre- and post-op: 27 (2.3) and 14 (4.6) mm Hg, respectively.[6,10] This can be explained by the observation that in our study, 64% of the patients had perforation peritonitis leading to elevated IAP which, after decompression and removal of liters of fluid and gas, returned to normal level immediately.

Though many of our patients at admission had elevated IAP along with multiple organ dysfunctions, they could not be identified as cases of primary ACS as the baseline data on their organ system functions were not available. In this subgroup of patients, associated renal dysfunction was seen in 78 (49%) patients and elevated IAP was found to have significant detrimental effect on blood urea, serum creatinine, and urine output. Sugrue *et al*. reported renal impairment in 20 (69%) patients of IAH.[6] There was no IAH seen in immediate post-op reading implying that no closure was done under tension.

At 6 hours post-op, seven patients were found to have IAH. Of these six had one or more newly developed organ system failure and hence a diagnosis of ACS was made in them. The IAP at this point was found to have significant detrimental effect on all the organ systems.

Applying McNemar statistics, role of decompression was studied in IAH patients. There was significant improvement seen in cardiovascular, renal and respiratory systems following laparotomy in patients who had pre-op IAH associated with organ system derangements. The mean (SD) pre- and post-op values of urine output in our pre-op IAH patients were 48.7 (13) and 53.9 (8.9) ml/hour, respectively, and that of serum creatinine were 1.5 (0.8) and 1.3 (0.6) mg/dl, respectively. We found significant improvement in the renal functions after laparotomy (χ^2^ = 13.33, *P* < 0.001). We also found a significant negative correlation between the IAH and urine output and a significant positive correlation between the IAH and serum creatinine. This is in agreement with the report by Ma *et al*. A significant negative correlation was reported between the IAH and urine output (*r* = –0.747, *P* < 0.01), and a significant positive correlation between the IAH and serum creatinine (*r* = 0.816, *P* < 0.01) by Ma *et al*. in 2005.[13] Sugrue *et al*. (1995) reported mean (SD) pre- and post-op values of urine output to be 1399 (617) and 1770 (870) ml/24 hours and that of serum creatinine to be 151 (86) and 128 (70) μmol/l, respectively.[6]

### Incidence

The incidence of IAH in our study was 80% at admission and 3.55% at 6 hours post-op. The incidence of post-op ACS was 3.05% in the general population, 13.16% in trauma patients, and 0.63% in non-trauma patients.

The incidence of IAH and ACS reported by various studies ranges from 2 to 78% and 0.5 to 36%, respectively, and depends on the population and the values used to define these entities.[14] The lower incidence observed was because this study includes lowrisk as well as high-risk patients, whereas most of the previous studies confined data collection to high-risk patients. While the latter approach ensures a good yield of patients with ACS, it may result in a very high incidence compared with that seen clinically in the general population overall. Furthermore, such an approach potentially misses those patients who are not at high risk, and yet may have Multiple Organ Dysfunction Syndrome (MODS) falsely attributed to sepsis or irreversible shock when in fact they have unrecognized ACS. By measuring the IAP prospectively in all patients, this study obtained true overall incidence. Also, an incidence of 3.05% in general population is significant enough to warrant further investigation in this group.

### Morbidity and mortality

No significant association was found between IAP at any point of time and occurrence of burst abdomen (*P* > 0.1). Also, preop IAP or IAP at 0 hours did not correlate significantly with the duration of hospital stay but IAP at 6 hours post-op was found to have a negative correlation (*r* = –0.234, *P* < 0.001) with the hospital stay, i.e., if IAP was elevated, duration of hospital stay decreased. R^2^ value (0.055) indicates that post-op pressure at 6 hours accounts for 5.5% of the total variance in duration of stay. With the help of the following regression equation, future estimation of duration of stay for the prospective patients with raised IAP can be calculated in a similar population:

Regression equation:

duration of stay = 11.238 + (−0.342 × post-op pressure at 6 hours).

This paradox was due to the finding that elevated IAP at 6 hours post-op invariably led to death and an early termination of hospital stay. After removing these IAH patients from the data, the analysis again showed no significant correlation (*r* = 0.075, *P* = 0.307) between IAP and duration of hospital stay.

Cheatham *et al*. had found that elevated IAP alone does not have sufficient sensitivity or specificity to be useful as a predictor of mortality.[11] But in our patients, elevated IAP pre-op, post-op at 0 and 6 hours was found to independently predict the occurrence of death (*P* < 0.001) but not at 24 hours (*P* = 0.35). However, a comparative view shows that odds of death are stronger as a result of increase in the post-op IAP at 6 hours in comparison with the pre-op or 0 hour post-op IAP. R^2^ value for post-op pressure at 6 hours is 0.173 which indicates that post-op pressure at 6 hours independently causes more than 17% of the total variance in the occurrence of death (rest of the 83% of the total variance in death is due to some other reasons). β coefficient value is 0.382 which shows that increase in each unit of postop pressure at 6 hours increases the probability of occurrence of death by a factor of 0.382 (provided the effect of all other factors is kept constant).

The mortality rate in pre-op IAH group was found to be 13.2% while that in ACS group was 100% despite decompression in three patients. In a retrospective study of patients with secondary ACS, overall mortality was 60% with 43% mortality for those decompressed.[15] The mortality despite decompression could have been due to early fulminant MODS or delay in decompression as the IAP readings were taken at 0 and 6 hours post-op with no reading in between. Hence, a more frequent IAP monitoring is recommended, at least in high-risk patients, as IAP measurement is simple and easy to perform. Also, it has high reproducibility and is minimally invasive.

The mortality associated with ACS as shown in various studies is compared in Table 8.[10–12,16–19] A similar mortality rate (100%) was seen in our study too when decompression was not done, supporting the view that ACS, if left untreated, is invariably fatal. Delayed decompression also has a high mortality rate indicating that a patient of ACS is salvageable only till the organ dysfunction is in a reversible phase. Hence, future studies on this subject should aim at devising a protocol which may help the healthcare professionals in early identification of the IAH and ACS patients and thus minimize the resulting high mortality.

**Table 8:** Reports of mortality associated with ACS

Authors	n	Decompression (%)	No decompression (%)
Kron *et al*.1984	11	28.6	100
Cullen *et al*. 1989	8	50	100
Fietsam *et al*. 1989	4	75	–
Morris *et al*.[17]	16	60	100
Bloomfield *et al*.[18]	1	0	–
Meldrum *et al*.[10]	21	28.6	–
Ivatury *et al*.[19]	23	34.8	–
Cheatham *et al*.[11]	73	64–68	89
Hong *et al*.[12]	6	50	–

*N*, NUMBER OF PATIENTS

## CONCLUSION

A total of 197 patients (149 men and 48 women) comprised the study population. The mean age of presentation was 34.78±14.9 (range 18–85) years with the most common age of presentation being 18 years. Most of the patients were of 21–40 years of age. The trauma cases comprised 19% of the study population with a predominance of penetrating injuries (60%) in this group. At admission, an overall 80% incidence of IAH was observed.

Oxygen saturation, urine output, and blood pressure were significantly lower (*P* < 0.05) in this subgroup, while blood urea and serum creatinine were significantly higher (*P* < 0.05) as compared to those who did not have IAH.

No significant association was found between IAP at any point of time and occurrence of burst abdomen (*P* > 0.1). Also, preop IAP or IAP at 0 hours did not correlate significantly with the duration of hospital stay but IAP at 6 hours post-op was found to have a negative correlation (*r* = –0.234, *P* < 0.001) with the hospital stay, i.e., if IAP was elevated, duration of hospital stay decreased due to increased mortality. With the help of the regression equation, future estimation of duration of stay for the prospective patients with raised IAP can be calculated in a similar population. This paradox was due to the finding that elevated IAP at 6 hours post-op invariably led to death in this study and an early termination of hospital stay. After removing these ACS patients from the data, the analysis again showed no significant correlation (*r* = 0.075, *P* = 0.307) between IAP and duration of hospital stay.

IAP pre-op, post-op at 0 and 6 hours was found to be a significant predictor of mortality in patients undergoing laparotomy (*P* < 0.001) but not at 24 hours (*P* = 0.35). While pre-op IAP as well as IAP at 0 hours post-op was significantly predictive, it was the IAP at 6 hours which showed the highest predictive value. Elevated IAP was found to affect all the organ systems adversely, and decompression led to an improvement in all the organ system functions as the IAP returned to normal value post-op.

The incidence of post-op ACS was 3.05% in the general population and 13.16% in trauma patients. The mortality rate for this subgroup was 100% in this study implying that a more frequent monitoring with prompt decompression is required to save these patients.

The following conclusions can be derived based on the study:

1. IAP is a significant predictor of mortality, but not of morbidity, in patients undergoing laparotomy
2. IAH has a detrimental effect on various organ systems.
3. Decompression leads to improvement in all the parameters.
4. The diagnosed cases of post-op ACS were associated with 100% mortality rate in this study.

A more frequent monitoring of IAP with prompt decompression may be helpful in decreasing this mortality rate. Further studies are required to establish a screening protocol in patients undergoing laparotomy to detect and manage cases of IAH and ACS.
